# Global incidence and trends of ocular cancer: A bibliometric analysis

**DOI:** 10.1016/j.aopr.2024.10.004

**Published:** 2024-10-28

**Authors:** Hang Xu, Alexander C. Rokohl, Xiaojun Ju, Yongwei Guo, Xincen Hou, Wanlin Fan, Ludwig M. Heindl

**Affiliations:** a Department of Ophthalmology, University of Cologne, Faculty of Medicine and University Hospital Cologne, Cologne, Germany; b Center for Integrated Oncology (CIO), Aachen-Bonn-Cologne-Dusseldorf, Cologne, Germany; c Eye Center, the Second Affiliated Hospital of Zhejiang University School of Medicine, Hangzhou, China

**Keywords:** Ocular cancer, Conjunctival cancer, Epidemiology, Incidence rate, Bibliometric analysis

## Abstract

**Background:**

Ocular cancer represents a significant threat to vision and life among various eye diseases. Conjunctival melanoma is regarded as one of the most feared and unpredictable ocular tumors. Considering the global differences in the occurrence of ocular melanoma across different races and regions, this study provides a thorough examination of the current state of research pertaining to the epidemiology of ocular and conjunctival cancers.

**Methods:**

This bibliometrics analysis used the Web of Science Core Collection (WoSCC) to collect data from publications on the epidemiology of ocular cancer, including relevant literature from 1951 to 2024. We examined indicators including t publication counts, citation rates, and data on contributing countries, institutions, and journals. Use VOSviewer and CiteSpace for network visualization and Microsoft Excel for data management. Our analysis reveals key trends in the epidemiology of ocular cancer across countries and identifies prominent keywords.

**Results:**

A total of 406 articles on ocular cancer were identified, with significant contributions from the United States, the United Kingdom, and Germany. Denmark also plays a crucial role, particularly in conjunctival cancer research. Carol L. Shields is a leading figure widely recognized for her influential citations in ocular cancer epidemiology. The top publication platforms include the *British Journal of Ophthalmology*, *Ophthalmic Epidemiology*, and *Ophthalmology*. Key terms in ocular cancer research focus on prevalence, survival, and epidemiology, while conjunctival cancer studies emphasize malignant melanoma, conjunctiva, and epidemiology. Through keyword co-occurrence and burst analysis, trending topics include prevalence, risk factors, uveal melanoma, choroidal melanoma, malignant melanoma, squamous cell carcinoma, and conjunctiva. For conjunctival cancer, key research areas expected to remain prominent are cell carcinoma, management, recurrence, ocular surface squamous neoplasia, and pathology.

**Conclusions:**

This analysis represents the first comprehensive bibliometric review mapping the trends and the knowledge structure in ocular cancer research, specifically from an epidemiological viewpoint. The results meticulously explore and encapsulate the research frontiers for scholars dedicated to the epidemiology of conjunctival cancer.

## 1 Introduction

Unlike other eye diseases, ocular cancer is a distinct one as it endangers vision and life. It is usually diagnosed from a detailed clinical history and specific ophthalmological tests by the treating clinicians.^1^^,^^2^^,^^3^ The University of Houston outlines the differences between primary malignant and secondary metastatic tumors in other areas or distant locations than those that spread from surrounding tissues.^2^ Types of Ocular Tumors Most commonly, Sarcomas, Lymphomas (HL and B cell), Malignant Eyelid Cancer with Basal Cell Carcinoma representing 90%, OSSN including dysplasia, carcinoma in situ SCC, Retinoblastoma, Choroidal Melanoma, Conjunctival melanoma (CoM), and Uveal Melanoma (UM).^1^^,^^2^^,^^4^^,^^5^ Among these, ocular melanoma is the most common primary eye cancer in adults, with approximately 83% arising in the uvea, 5% originating intraconjunctivally, and around 10% developing elsewhere in an eye.^6^ Conjunctival melanoma, one of the ocular surface tumors accounting for its malignant and aggressive character, scores the highest, with a local recurrence rate close to 50% within ten years after the primary treatment.^7^ This malignancy can be locally invasive and spreads systemically via both lymphatic and hematogenous pathways, with a risk for recurrence following treatment.^8^

Northern Europe and Australia have the highest rates, whereas there are lower incidences in Asian, Hispanic, and Black populations of ocular melanoma.^6^ It affects predominantly white males and other immunosuppressed individuals.^9^ Initial research showed that certain types of melanoma and ocular melanomas, in general, are rare diseases with incidences among white populations, though racial or regional differences may exist. The available literature suggests that ocular melanoma occurs more frequently among Caucasians and less so for other racial groups.^10^ ; however, limited numbers of population-based incidence studies have been conducted on occurrence rates in Asian populations.

Bibliometrics is a quantitative study of scientific publications. The total item is Science Bibliometric Studies. Methods like co-word, social network, and cluster analysis were used to get a general perspective of the development trend. Analysis method: Using statistical analysis, authors, journals, and institutions identified key areas and trends.^11^ By the co-citation analysis on visualization tools, the bibliometric study creates a knowledge domain map to show research topics and their relations.^12^

Previous bibliometric studies on ocular cancer have mainly focused on tumor treatment, broad cancer categories, or individual tumors, leaving a gap in the epidemiology of ocular tumors. For example, Christophe Boudry et al. (2015)^13^ analyzed global trends in eye neoplasms, and Xu et al. (2022)^14^ studied conjunctival melanoma; however, neither study delved deeply into tumor incidence patterns. Our study addresses a critical gap in ocular oncology research by analyzing regional disparities in research output and their impact on global trends. We reveal significant variations in research productivity and collaboration patterns across regions, offering a nuanced perspective on the global landscape of ocular tumor epidemiology. This analysis highlights underrepresented areas, emphasizing the need for increased local research and international collaboration. Our comprehensive bibliometric study provides valuable insights for researchers and clinicians in ocular and conjunctival cancer epidemiology by identifying current trends and potential future directions.

## 2 Material and methods

### 2.1 Data collection and data retrieval strategy

This cross-sectional study collected publication data on April 17, 2024 and downloaded in "plain text" format from WoSCC.

The retrieved publications had to meet the following criteria.

(1) The search terms for total ocular/conjunctival cancer were determined using the TS ("topic") function, which includes the title, abstract, author keywords, and keywords Plus. The search criteria for the ocular cancer were defined as TS = (ALL= (eye cancer) OR ALL=(ocular melanoma) OR ALL=(ocular neoplasms) OR ALL=(eye tumor) OR ALL=(ocular tumor) OR ALL=(ocular cancer)) AND (ALL=(epidemiology) OR ALL=(incidence rate)); The search criteria for total conjunctival cancer were defined as TS = (ALL=(conjunctival cancer) or ALL=(conjunctival melanoma) or ALL=(conjunctival neoplasms) or ALL=(conjunctival tumor) or ALL=(conjunctival tumor) or ALL=(conjunctival squamous cell carcinoma) or ALL=(ocular surface squamous neoplasia)) AND (ALL=(Epidemiology) or ALL=(incidence rate);
(2) the document type was "article";
(3) Unlimited publication period;
(4) the following information was collected: publication, authors, countries, institutions, journals, keywords, and citations.

The only difference between the search for articles on ophthalmic tumors and that on conjunctival tumors is the search keywords in the first step; the rest of the steps are the same.

### 2.2 Data analysis

This study employs bibliometric methods alongside various analytical tools to comprehensively analyze the research field. We extracted bibliographic data from the Web of Science (WOS) database and saved it as a TXT file named "XXX". The dataset encompasses publication counts, citation frequencies, and information regarding countries, institutions, and journals. The primary visualization tools utilized are VOSviewer (version 1.6.17) and CiteSpace (version 6.3.R2), with Microsoft Excel employed for data processing and chart generation. Excel was used to create charts that illustrate the research performance of top-ranking countries, institutions, authors, and journals. This multidimensional, multi-tool analytical approach enables a comprehensive understanding of the research domain's developmental dynamics, collaboration patterns, and emerging topics. VOSviewer was employed to extract and process bibliometric data, generating visual maps of country collaboration networks and high-frequency keyword networks. CiteSpace was utilized for visualizing keyword temporal trends and burst detection, with the analysis period spanning from 1951 to 2024, despite the first relevant literature being published in 1996. The time-slicing was set to three years, the Top N threshold was established at 30, and the g-index option set the k value to 25. Citation analysis was performed using the Pathfinder and Pruning sliced networks methods, with the node type designated as keywords. We simplified the network structure by applying the Pathfinder and pruning algorithms to individual and merged networks while maintaining all other parameters at their default settings. This multi-dimensional strategy offers a comprehensive perspective on the evolution and present condition of the field.

## 3 Results

### 3.1 Overall publication trends

**Fig. 1:**
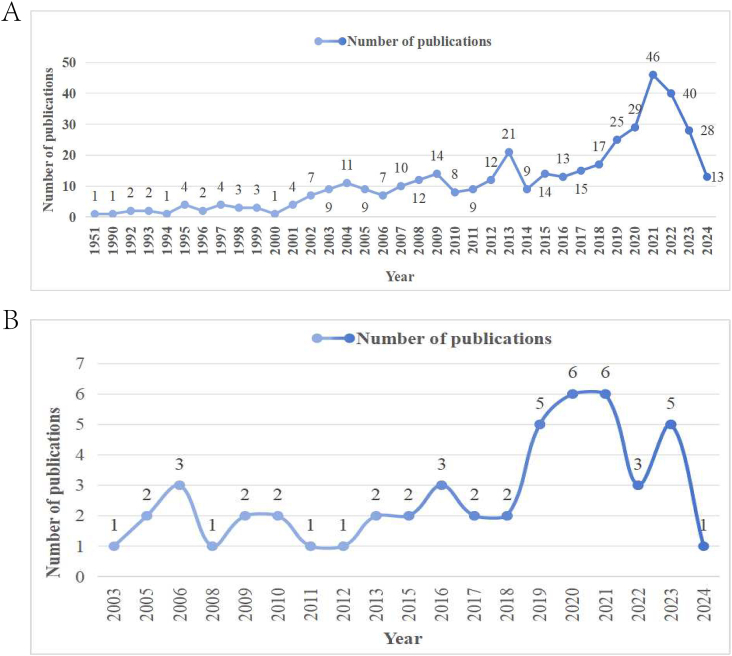
(A)The annual publications on epidemiology in ocular cancer between 1951 and 2024, (B) the annual publications on epidemiology in conjunctival cancer between 2003 and 2024.

Quantitative analysis of published papers can help identify the most influential and valuable contributions to the academic community. This information can enhance the quality of scientific literature and guide researchers in their investigative pursuits. Fig. 1 (A and B) illustrates the annual publication output concerning ocular and conjunctival cancer. From the epidemiological literature, 406 articles on ocular cancer were retrieved from 1951 to 2024, while 50 articles on conjunctival tumors were identified from 2003 to 2024. The number of epidemiological articles on ophthalmic tumors from 1951 to 2000 exhibited a relatively slow overall trend, with occasional increases in individual years. However, from 2000 to 2024, a clear growth trend is evident, peaking at 46 articles in 2021, followed by a noticeable decline from 2021 to 2024. In contrast, the volume of epidemiological articles on conjunctival cancer has remained relatively stable from 2003 to 2024, although notable peaks in publications occurred in 2020 and 2021.

### 3.2 Analysis of published articles by countries/regions and institutions

**Fig. 2:**
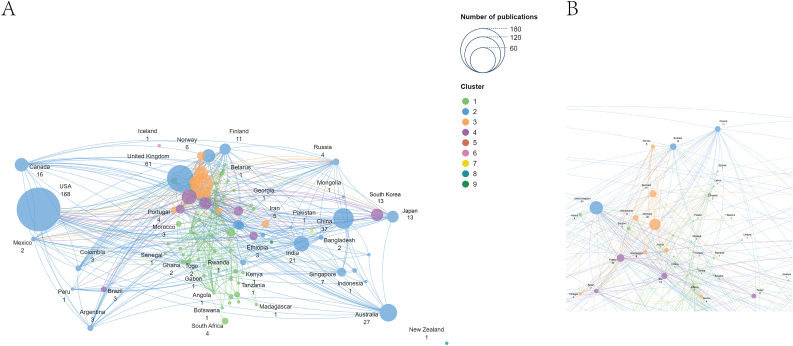
Network visualization map for country collaboration for ocular cancer.

Ocular cancer is a subject of global significance, as evidenced by the publication of 406 papers across 71 academic journals authored by 2821 researchers from 1021 different institutions in 239 countries and regions, which contributes to epidemiological studies, as shown in Fig. 2. Fig. 2 highlights global research collaboration in ocular cancer, with high-income countries in North America, Europe, and East Asia leading. The network is divided into five clusters, with three main ones: cluster 1 (red) includes the USA, Canada, and the UK, showing strong collaboration among high-income countries; cluster 2 (blue) consists of China, Japan, and Australia, representing partnerships in East Asia and the Asia-Pacific; and cluster 3 (green) features European countries like Germany and Italy, reflecting close ties within Europe.

**Fig. 3:**
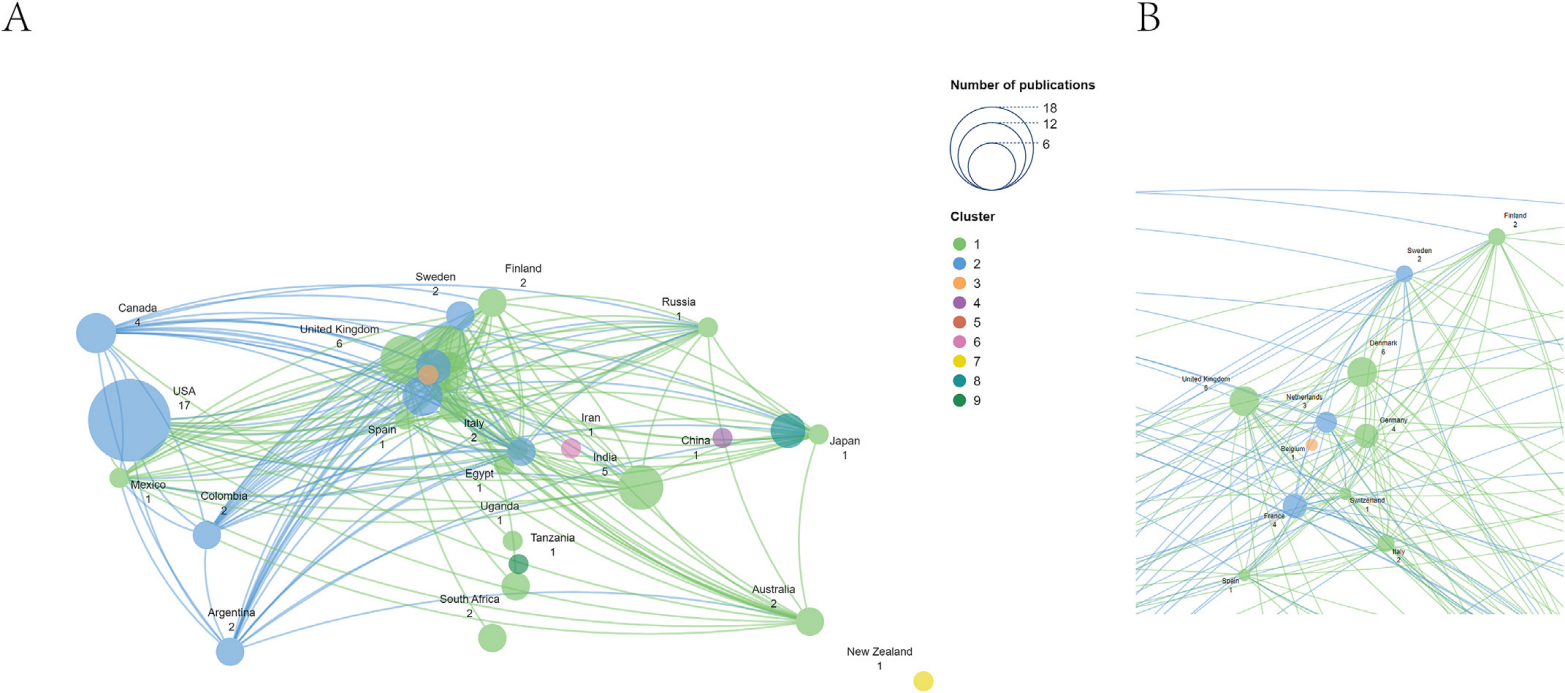
International Collaboration Networks in Conjunctival Cancer Epidemiology Research (A) Global collaboration network (B) Focused view of European collaborations.

Table 1 displays the top 10 countries with the highest research output. Regarding national research capabilities, the USA is at the forefront with 167 publications and 7664 citations, followed by the UK, which has 60 publications and 1988 citations. In comparison, Germany follows with 44 publications and 1051 citations. An examination of collaboration from 1951 to 2024 reveals that the USA boasts the most extensive international partnerships in this area, with the UK maintaining the strongest ties to the USA. Specifically regarding conjunctival cancer, a total of 50 papers published in 21 academic journals were produced by 291 researchers from 228 institutions across 62 different countries and regions. Table 2 enumerates the ten countries with the highest publication totals. A co-authorship network was created for countries and regions with at least one published paper (T ​= ​1) (Fig. 3A and B). The visual representation depicts the level of collaboration among these nations and regions, with the USA identified as the largest node within the national network map (Fig. 3A). Again, the USA holds the top position in terms of national research output with 17 publications and 539 citations.

**Table 1:** The top 10 countries/regions that publications on epidemiology in ocular cancer.

Countries/Regions	Documents	Citations	Total Link Strength
United States	167	7664	122
United Kingdom	60	1988	133
Germany	44	1051	75
China	37	405	50
Australia	27	684	70
India	21	916	56
France	19	821	94
Canada	16	320	58
Denmark	16	534	44
Sweden	14	454	50

**Table 2:** The top 10 countries/regions that publications on epidemiology in conjunctival cancer.

Countries/Regions	Documents	Citations	Total Link Strength
United States	17	539	28
Denmark	6	269	21
United Kingdom	6	178	32
India	5	60	20
Canada	4	95	28
France	4	83	28
Germany	4	66	22
Netherlands	3	211	28
South Korea	3	50	0
Argentina	2	56	28

Denmark and the UK each contributed six publications, differing in citation counts (269 and 178, respectively). Moreover, the top 10 most productive institutions are outlined in Table 3, with Harvard University leading with 16 publications and 1784 citations, followed by Thomas Jefferson University with 14 publications and 1267 citations, and Moorfields Eye Hospital NHS Foundation Trust, which also has 14 publications but 684 citations. When setting a minimum publication threshold for institutions, 46 institutions satisfied the criteria.

**Table 3:** The top 10 institutions with the most published articles on epidemiology in ocular cancer.

Organization	Documents	Citations	Total Link Strength
Harvard Univ	16	1784	14
Thomas Jefferson Univ	14	684	32
Moorfields Eye Hosp Nhs Fdn Trust	14	1267	5
Thomas Jefferson Univ	14	428	14
Univ Melbourne	13	550	14
Lv Prasad Eye Inst	12	413	26
Ucl	10	635	28
Ucl Inst Ophthalmol	10	337	16
Univ Copenhagen	9	92	7
Harvard Med Sch	9	448	27
Univ Cambridge	9	180	0

Overall, the USA and the UK play pivotal roles in researching the epidemiology of ocular and conjunctival cancer. Germany is particularly influential in ocular cancer research, while Denmark is prominent in conjunctival cancer research.

### 3.3 Analysis of authors, co-cited authors, journals and co-cited journals in ocular cancer

The bibliometric analysis of ocular cancer literature revealed 406 articles authored by 2061 unique researchers, with a total of 8215 author appearances. The mean authorship per publication was 5.0, indicative of substantial collaborative efforts in this field. Table 4 presents the top 10 most prolific authors in the field, whose collective output of 77 publications represents 18.96% of the total literature. This concentration of authorship highlights the significant contributions of key researchers to the domain's knowledge base. The most productive authors were Carol L. Shields [10 publications (2.46%)], followed by Paul T. Finger [9 publications (2.21%)], Steffen Heegaard [9 publications (2.21%)], Swathi Kaliki [9 publications (2.21%)], and Jerry A. Shields [8 publications (1.97%)]. Table 5 lists the ten most frequently co-cited authors, including Carol L. Shields (235), Arun D. Singh (104), Paul T. Finger (85), Jerry A. Shields (85), and Johanna M. Seddon (59). Carol L. Shields, Paul T. Finger, Swathi Kaliki, Arun D. Singh, and Jerry A. Shields are among the top 5 authors in both lists, indicating their significant influence in the field.

**Table 4:** The top 10 most productive authors that published papers on epidemiology in ocular cancer.

Author	Documents	Citations	Total link strength
Shields, Carol L.	10	324	11
Finger, Paul T.	9	387	5
Heegaard, Steffen	9	276	5
Kaliki, Swathi	9	239	4
Shields, Jerry A.	8	278	11
Foster, Paul J.	7	155	33
Singh, Arun D.	7	946	1
Bornfeld, Norbert	6	43	0
Dalvin, Lauren A.	6	36	2
Khaw, Kay-Tee	6	145	32
Khawaja, Anthony P.	6	135	31
Luben, Robert	6	145	32

**Table 5:** The top 10 co-cited authors with the most publications on epidemiology in ocular cancer.

Author	Citations
Shields, Cl	235
Singh, Ad	104
Finger, Pt	85
Shields, Ja	85
Seddon, Jm	59
Kaliki, S	47
Abramson, Dh	43
Klein, R	43
Albert, Dm	41
Damato, B	39

Table 6 illustrates that 58.62% (n ​= ​238) of the field's publications are concentrated in ten journals. The *British Journal of Ophthalmology* leads with 47 publications, followed by *Ophthalmic Epidemiology* (44), *Ophthalmology* (37), *American Journal of Ophthalmology* (28), and *Eye* (22). Journal influence, as measured by co-citation frequency, is presented in Table 7. The top five co-cited journals in ocular cancer epidemiology are *Ophthalmology* (1216 co-citations), *Archives of Ophthalmology* (759), *British Journal of Ophthalmology* (694), *American Journal of Ophthalmology* (589), and *Investigative Ophthalmology & Visual Science* (466). This distribution highlights the primary publication venues and the most influential sources in the field.

**Table 6:** The top 10 journals that published papers on epidemiology in ocular cancer.

Source	Documents	Citations	Total link strength
British Journal Of Ophthalmology	47	1355	1433
Ophthalmic Epidemiology	44	828	1073
Ophthalmology	37	3812	1100
American Journal Of Ophthalmology	28	1963	748
Eye	22	747	899
Investigative Ophthalmology & Visual Science	19	981	857
Acta Ophthalmologica	12	405	473
Ocular Oncology And Pathology	11	82	688
Archives Of Ophthalmology	9	757	205
Journal Francais D Ophtalmologie	9	96	134

**Table 7:** The top 10 co-cited journals with the most publications on epidemiology in ocular cancer.

Source	Citations	Total link strength
Ophthalmology	1216	32156
Arch Ophthalmol-Chic	759	21296
Brit J Ophthalmol	694	21733
Am J Ophthalmol	589	19653
Invest Ophth Vis Sci	466	16822
Eye	201	5623
Surv Ophthalmol	176	4792
Acta Ophthalmol	159	4852
Jama Ophthalmol	145	3597
Lancet	127	3288

This bibliometric analysis highlights the leading authors, journals, and research trends in ocular cancer epidemiology. The USA and the UK play pivotal roles in the research, with significant contributions from Germany in ocular cancer and Denmark in conjunctival cancer.

### 3.4 Analysis of references with citation burst in ocular cancer

Reference co-citation is a method to measure the degree of association between references. Specifically, two papers (or multiple papers) are cited simultaneously by one or more subsequent papers, forming a co-citation relationship. CiteSpace can partition a co-citation network into distinct clusters, grouping closely associated references together while segregating loosely connected references into separate clusters.

**Fig. 4:**
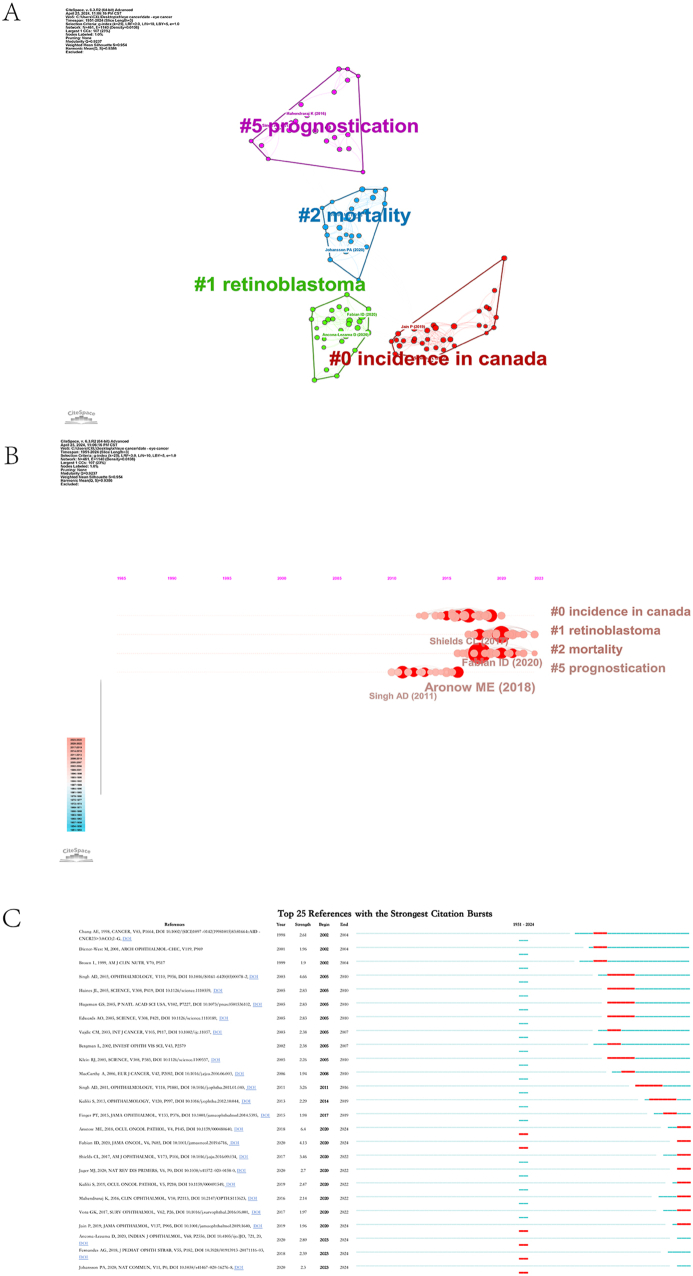
(A) Clustered network map of co-cited references (B) Timeline view of reference clusters, ordered by size, with nodes indicating their first appearance and connections representing co-citations. (C) Top 25 references with the highest citation.

As shown in Fig. 4A, the co-citation network can be segmented into four significant subclusters. The evaluation indicators of the clustering results are the clustering modularity (Q value) and the clustering mean profile (S value). When Q ​> ​0.3, it means that the clustering structure is significant, and when S ​> ​0.7, it means that the clustering theme is prominent and the article similarity is high. The results in the figure show that Q ​= ​0.9237 and S ​= ​0.954, indicating that the clustering results are meaningful and compelling. There are 461 nodes and 1140 edges, forming four clusters.

Clusters with smaller numbers encompass more nodes. The four primary clusters identified from the cited literature are #0 incidence in Canada, #1 retinoblastoma, #2 mortality, and #5 prognostication (Fig. 4A). Cluster 4 was excluded due to an insufficient number of articles. These clusters can be presented in a timeline format, with cluster numbers on the y-axis, to depict the temporal evolution of research hotspots (Fig. 4B). This timeline plot demonstrates the field's research progression and four subfields.

Table 8 lists the top 10 most cited original publications on the epidemiology of ocular cancer from 1998 to 2018. The most frequently cited paper is by Singh AD, published in 2011 in *Ophthalmology,* with 23 citations and a total link strength of 270, followed by another paper by Singh AD from 2003 in the same journal, with 21 citations and 261. The third most cited paper, authored by Kujala E in 2003 and published in *Investigative Ophthalmology & Visual Science*, received 20 citations and a total link strength of 261. These papers highlight key research trends and contributions to ocular cancer epidemiology over the past two decades.

**Table 8:** The top 10 papers with most citations on epidemiology in ocular cancer.

Paper	DOI	Citations	Total link strength
SINGH AD, 2011, OPHTHALMOLOGY	10.1016/j.ophtha.2011.01.040	23	270
SINGH AD, 2003, OPHTHALMOLOGY	10.1016/s0161-6420(03)00078-2	21	261
KUJALA E, 2003, INVEST OPHTH VIS	10.1167/iovs.03-0538	20	261
CHANG AE, 1998, CANCER	10.1002/(sici)1097-0142(19981015)83:8 ​< ​1664:aid-cncr23 ​> ​3.0.co; 2-g	18	218
ARONOW ME, 2018, OCUL ONCOL PATHOL,	10.1159/000480640	17	166
BERGMAN L, 2002, INVEST OPHTH VIS	NA	15	198
KIVELÄ T, 2009, BRIT J OPHTHALMOL	10.1136/bjo.2008.150292	15	158
VIRGILI G, 2007, OPHTHALMOLOGY	10.1016/j.ophtha.2007.01.032	15	182
DIENER-WEST M, 2001, ARCH OPHTHALMOL-CHIC	NA	14	155
YU GP, 2003, AM J OPHTHALMOL	10.1016/s0002-9394(02)02288-2	14	169

Citation bursts, indicative of heightened scholarly attention during specific periods, offer insights into research trends and impact.^15^ Fig. 4C uses a color-coded temporal map to illustrate the 25 most significant citation bursts from 2002 to 2024. In this visualization, light blue denotes the absence of activity, dark blue represents low-intensity citations, and red signifies high-frequency bursts. This bibliometric phenomenon reflects temporal citation pattern shifts, elucidating the field's evolutionary trajectory. The initial citation burst emerged in 2002, stemming from Chang AE et al., 's 1998 publication.^11^ Notably, the most intense recent bursts originated from Aronow ME et al. (2018),^16^ closely followed by Singh et al. (2003).^17^ These findings underscore the dynamic nature of influential research within the field, highlighting seminal works and contemporary contributions that have significantly shaped scholarly discourse. The latest outburst began in 2020 and has continued for four years.

### 3.5 Analysis of keywords and hotspots

Keywords provide researchers with valuable insights into the research topics and methods of publications. Keyword co-occurrence analysis aims to study the relationships between frequently appearing keywords in publications, reflecting research hotspots.

In our study on ocular cancer, we analyzed 1778 keywords from 406 articles using VOSviewer. Keywords that appeared more than three times in the titles and abstracts were included, resulting in 249 keywords after excluding 12 unrelated terms, such as "dependent probe amplification" and "experience". The co-occurrence of keywords, indicating their appearance in the same publication, provides better insights into research hotspots than single keywords.

Key terms like epidemiology (155), prevalence (48), survival (46), risk factors (40), and cancer (39) were central in the visualization network map. VOSviewer grouped all keywords into six clusters, with the top three represented in Figure A: red (cluster 1: "epidemiology"), green (cluster 2: "survival"), and blue (cluster 3: "cancer"). The main keywords in the "epidemiology" cluster included prevalence, progression, and risk factors. In the "survival" cluster, key terms were uveal melanoma, choroidal melanoma, and malignant melanoma. The "cancer" cluster focused on keywords like conjunctiva, tumors, and squamous-cell carcinoma.

**Fig. 5:**
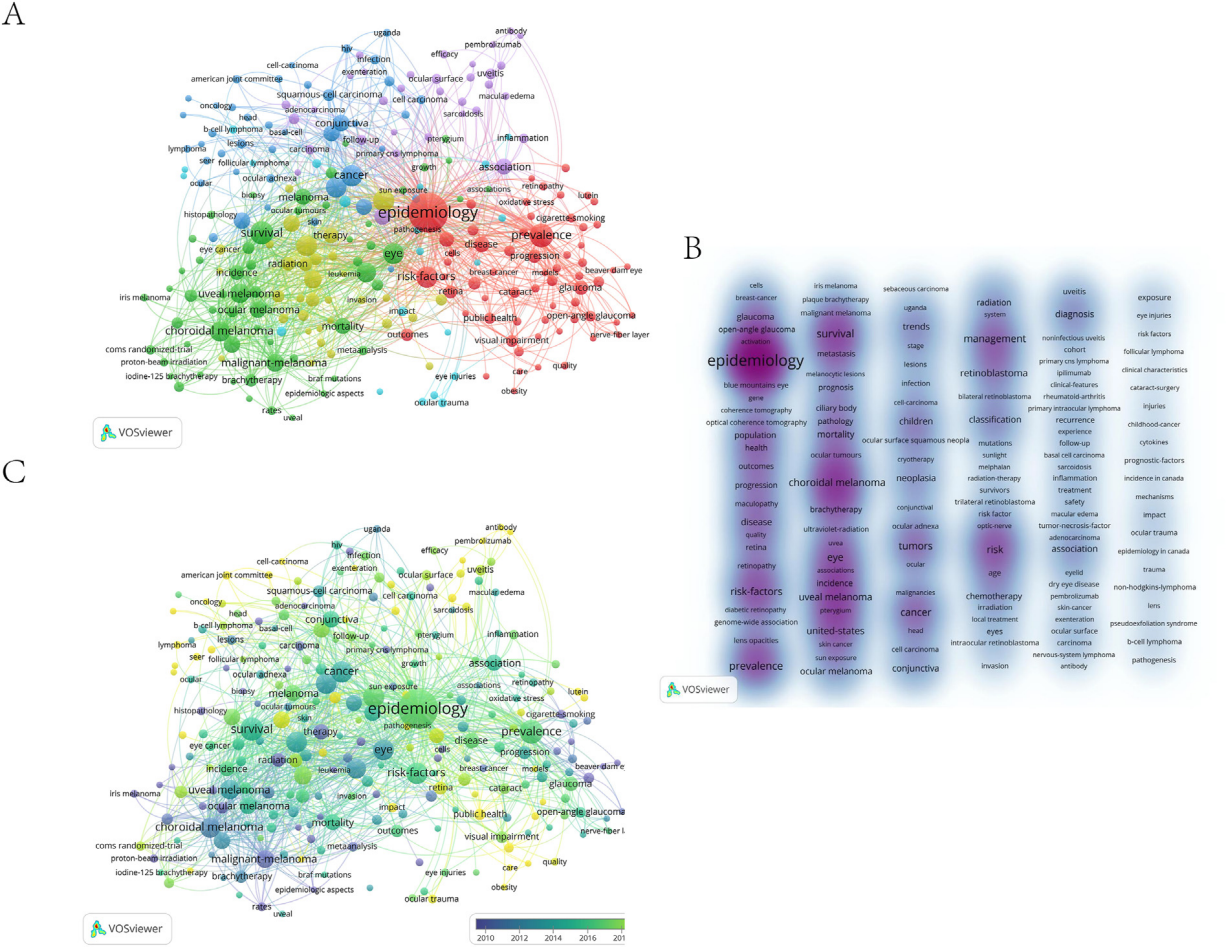
Keyword Analysis in Ocular Cancer (A) Co-occurrence network of keywords visualized using VOSviewer (B) Density visualization of keyword distribution (C) Temporal evolution of keywords represented through overlay visualization.

To highlight the evolution of research hotspots, we presented a time-zone diagram of the keyword co-occurrence network (Fig. 5B and C). The density visualization (Fig. 5B) showed "epidemiology", "prevalence", and "survival" as the most frequent keywords. The overlay visualization map (Fig. 5C) summarized keyword occurrences over time, providing a temporal perspective on research trends in ocular cancer.

**Fig. 6:**
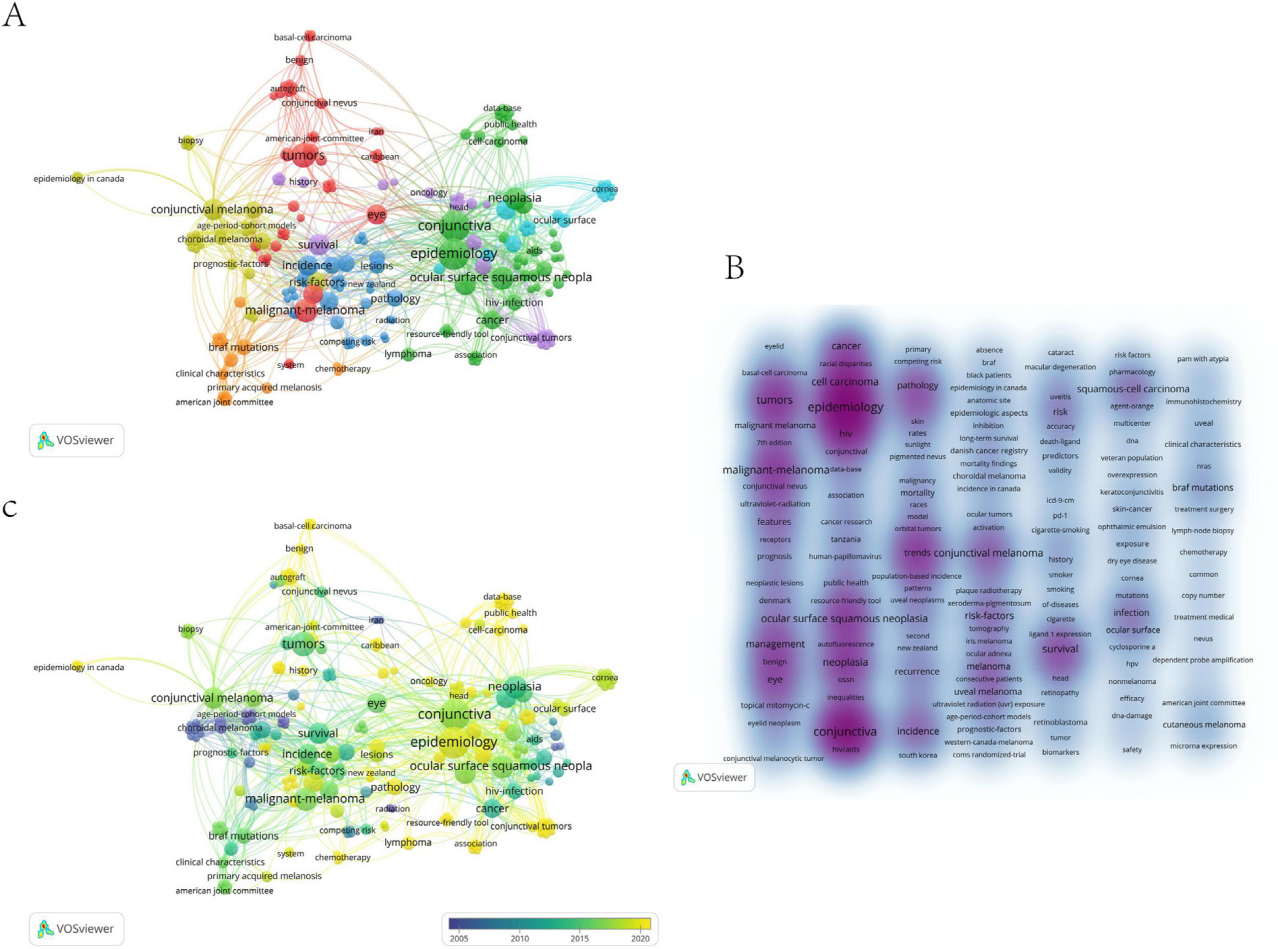
Keyword Analysis in Conjunctival Cancer (A) Co-occurrence network of keywords visualized using VOSviewer (B) Density visualization of keyword distribution (C) Temporal evolution of keywords represented through overlay visualization.

Focusing on the incidence of conjunctival tumors, we analyzed 235 keywords from 50 publications using VOSviewer. Keywords appearing more than once included epidemiology (19), conjunctiva (18), malignant melanoma (11), neoplasia (10), and ocular surface squamous neoplasia (9), which were central in the visualization network map. VOSviewer grouped these keywords into six clusters, with the top three clusters being red (cluster 1: epidemiology"), green (cluster 2: "tumors"), and blue (cluster 3: "trends") (Fig. 6A). The main keywords in the "epidemiology" cluster were conjunctiva, cell carcinoma, and ocular surface squamous. The "tumors" cluster included malignant melanoma, management, and eye. The "trends" cluster featured keywords like incidence, recurrence, and pathology. To track research hotspots over time, we created a time-zone diagram of the keyword co-occurrence network (Fig. 6B and C).

## 4 Discussion

This study illustrates the annual publication trends in ocular cancer (1951–2024) (Fig. 1A) and conjunctival cancer (2003–2024) (Fig. 1B), highlighting a recent surge in interest in these fields. The USA emerges as the most prolific country in ocular and conjunctival cancer research, boasting the highest number of publications and the most significant total citation frequency, as shown in Table 1, Table 2, and Fig. 2, Fig. 3. Fig. 2 showcases the global research collaboration network in ocular cancer, emphasizing the leadership of high-income countries in North America, Europe, and East Asia. In contrast, countries in South America and Africa show relatively fewer research partnerships, reflecting disparities in research output and resources in these regions. The USA's leadership in this domain is attributable to its top-tier researchers and institutions, which play vital roles in epidemiological investigations of ocular cancer.

Notably, researchers like Shields and Carol L. have significantly contributed to the citation landscape of epidemiological studies in ocular cancer. These findings underscore the potential impact of intensified collaborative research, which could drive significant advancements in the field. Such partnerships are poised to yield impactful studies bolstered by solid leadership and substantial financial support from productive countries. Table 4 showcases authors with numerous publications at the forefront of ocular cancer research, now focusing on recent innovations. The *British Journal of Ophthalmology* is the most frequent publication venue, followed by *Ophthalmic Epidemiology* and *Ophthalmology*. Given the rarity of ocular cancer, limited journals are dedicated to this niche, directing researchers to these critical sources for the latest epidemiological developments. Future researchers are encouraged to target these top journals to disseminate their significant findings.

Research frontiers in a specific academic field can be elucidated through keyword co-occurrence analysis. Using data from WoSCC, keywords were categorized into six clusters, with the most prominent three being "epidemiology", "survival", and "cancer". These clusters highlight the primary research trajectories within the epidemiological study of ocular cancer. The overlay visualization in Fig. 5C reveals a shift in research focus from "epidemiology" to "pathogenesis", "risk factors", and "therapy", with specific attention to the progression from "uveal melanoma" to "choroidal melanoma" and "iris melanoma".

In the case of conjunctival cancer, as shown in Fig. 6C, research emphasis has shifted from "epidemiology" to "pathology", "chemotherapy", and "radiation". This change reflects broader trends in medical research, highlighting the continued focus on identifying and addressing risk factors for ocular tumors.

Analyzing the progression of morbidity in different regions is crucial for identifying potential risk factors, thereby improving our understanding and management of disease mechanisms. Advances in public health and basic scientific research have resulted in significant breakthroughs in classifying and determining the incidence rates of eye tumors in different regions. Furthermore, an examination of keywords with the highest increase in research interest suggests that topics such as "BRAF mutations", "brachytherapy", and "oxidative stress" are likely to become critical areas of focus in future research efforts.

Uveal melanoma, a malignant tumor of the uvea, is the most common intraocular neoplasm in adults.^18^ The incidence rates of uveal melanoma remained almost constant between 1983 and 1994 in Europe.^19^ In the USA, an age-adjusted incidence rate of 5.1 per million was unchanged from 1973 to 2008, and during that time survival for these patients did not show improvement.^20^ Moreover, a 5-year update showed that the age-adjusted incidence of uveal melanoma in the USA (5.2 per million) remained stable, although only a slight increase was noted in the whites between 2008 and 2013.^21^ Throughout the study, metastatic uveal melanoma remained the leading single cause of death in most cases.^22^ Genetics and immunology will be cutting-edge research areas in uveal melanoma,^12^ with attention to genes such as GNAQ, GNA11, and BAP1, highlighted as important focus points.^18^

According to a report by Vega-Escobar et al., based on a study conducted between 1962 and 2019 in Cali, Colombia, the ocular neoplasms that were most prevalent included retinoblastoma (21%), squamous cell carcinoma (20%), melanoma (16%), and lymphoma (8%).^23^ RB is the most common malignant disorder of childhood, which can lead to death when overlooked.^24^ It accounts for roughly 11% of all cancers diagnosed within the first year of life, with 95% of club RB diagnosed before the age of five.^2^ Its malady becomes manifest through the inactivation of both alleles of the RB1 gene.^25^ Though hereditary factors are very important, other factors such as environmental and lifestyle features have also been contributing causes of the sporadic cases, especially in resource-poor settings.^26^

Conjunctival tumors are common, with ocular surface squamous epithelial neoplasms, melanomas, and lymphomas being the most significant malignancies.^27^ Conjunctival malignant melanoma (CoM), a pigmented ocular surface lesion,^8^ redominantly affects individuals over 50, particularly in White populations.^28^ Unlike the rising incidence of cutaneous melanoma (CM), CoM incidence has stabilized and decreased in the U.S. from 2000 to 2020.^29^ Conversely, Australia reported an increased rate of CoM diagnoses from 1982 to 2014, with stable disease survival at 90%.^30^ These varying trends may reflect healthcare access disparities and risk factors across income levels.^29^ Similar to cutaneous melanoma but distinct from uveal melanoma, CoM has been shown to harbor UV radiation-associated genetic mutations.^31^ This connection to UV radiation is particularly relevant for regions like South Africa, which, despite being in the mid-latitudes, experiences high levels of sunshine, with a summer UV index frequently exceeding 10. Given these UVconditions, populations with fair skin are at a heightened risk of developing skin cancer, which supports the idea that UVexposure may also play a significant role in the etiology of CoM in such regions.^32^ Other risk factors include TP53 gene alterations, immunosuppression, vitamin A deficiency, xeroderma pigmentosum, allergic conjunctivitis, smoking, and chemical exposure.^33^ Current genetic research emphasizes the similarities between CoM and CM, with successful CM treatments becoming a focus for CoM research.^14^

This bibliometric study provides valuable data contributing to a better understanding of the research field in eye neoplasms. By highlighting key trends, influential researchers, and emerging areas of interest, we can better target future research efforts and improve outcomes for patients affected by these conditions.^13^ To our knowledge, this type of bibliometric analysis related to ophthalmic and conjunctival tumors has never been performed before, and no network visualization map of national collaborations has been applied in the field of ophthalmic tumor epidemiology. The WoS database used in this study, as opposed to PubMed, which we have done in the past and was also used for bibliometric analysis, can quantify article citations and provide a qualitative assessment.

This study has limitations that warrant consideration. our search was limited to the Web of Science database, which may result in selection bias towards English-language publications that potentially exclude significant contributions from non-English speaking regions or grey literature. Beyond uncovering relevant trends, the period of choice may be insufficient to illustrate major long-term ocular cancer research themes fully. This is mainly due to superior research funding in countries like the USAand UKcompared with others, combined with advanced healthcare systems that generate vast amounts of clinical data — which are often shared through international collaborations. These elements elevate their research impact and international presence in ocular oncology. Therefore, we suggest broadening the scope of the study by including more databases, specifically non-English publications, and extending the timeframe to incorporate long-term trends. Obscure parts, mainly in the southern hemisphere, would show how geographic and demographic factors impact the incidence of ocular cancer. A detailed study analyzing research trends from those regions and fostering international collaborations can close knowledge gaps and give more inclusive insights. These approaches would present a more holistic and equitable perspective on global disparities in ocular cancer research and advance this vital field.

## 5 Conclusions

This study has illuminated global trends in ocular and conjunctival cancer research, with a focus on epidemiology. Our findings reveal that the USA leads in research contributions, while journals such as *British Journal of Ophthalmology* and *Ophthalmology* have shown significant progress. Carol L. Shields emerges as a prominent figure for potential academic collaboration. Our analysis demonstrates that epidemiology and survival have been the primary focus in ocular cancer research, whereas conjunctival cancer research has concentrated on epidemiology and malignant melanoma. Through keyword co-occurrence analysis, we identified "epidemiology", "prevalence", and "survival" as the most frequent keywords. Moreover, the timeline visualization indicates a gradual shift in research focus from epidemiology towards pathogenesis, risk factors, and therapy.

Results yielded during this study may be beneficial for devising clinical practices and public health efforts. New agnostic entities like BRAF, as well as old and new trends of management such as the use of brachytherapy, overcoming hypoxia-induced resistance to radiation therapy by targeting oxygen metabolism, e.g., through inhibition autophagy/microbial ATP synthesis pathways could be attempted. Our study, therefore, highlights which regions are still underrepresented and for whom we should promote additional focus in public health. Moreover, this study points out the need to resolve disparities created by research gaps. Well-known research-productive countries such as the USA can be seen to lead in learning how to efficiently spend money and increase international collaboration. As a result, policy-making efforts ought to focus on fair research and prayer efficiency by ensuring the resources are channeled toward the high-burden areas for an improved overall global outlook. In the future, subsequent studies should attempt to investigate more specific eye cancer subsites and their incidence, mainly in areas where this type of neoplasm is not sufficiently represented. However, further studies will be necessary to explore the role of genetic and environmental risk factors. Ultimately, global collaboration will be necessary to ensure that the progress associated with research benefits patients globally.

To our knowledge, this bibliometric study provides a complete view of ocular and conjunctival cancer research. It points out directions for future investigations hidden in current evidence and emphasizes integration strategies that are essential for more comprehensive global representation.

## Study approval

Not Applicable.

## Author contributions

The authors confirm contribution to the paper as follows: conception and design of study, data collection: WF, ACR, MD; analysis and interpretation of results, drafting: HX. Drafting the manuscript: XJ, YG, XH; All authors reviewed the results and approved the final version of the manuscript.

## Funding

This work was supported by China Scholarship Council(item CSC No.202208330084).

## Declaration of competing Interest

The authors declare that they have no known competing financial interests or personal relationships that could have appeared to influence the work reported in this paper.

## Abbreviations

WoSCC: Web of Science Core Collection
BCC: Basal Cell Carcinoma
OSSN: Ocular Surface Squamous Neoplasia
SCC: Squamous Cell Carcinoma
RB: Retinoblastoma
UM: Uveal Melanoma
CoM: Conjunctival Melanoma
